# (4R*,5R*)-Diethyl 2-(4-nitro­phen­yl)-1,3-dioxolane-4,5-dicarboxyl­ate

**DOI:** 10.1107/S160053681201118X

**Published:** 2012-03-21

**Authors:** Chun-Lei Lv, Jian-Hui Chen, Yu-Zhe Zhang, Ding-Qiang Lu, Ping-Kai OuYang

**Affiliations:** a School of Pharmaceutical Science, Nanjing University of Technology, Xinmofan Road No. 5 Nanjing, Nanjing 210009, People’s Republic of China; b Xinchang Pharmaceutical Factory, Zhejiang Medicine Co. Ltd, Xinchang 312500, People’s Republic of China; c College of Materials Science and Engineering, Nanjing University of Technology, Xinmofan Road No. 5 Nanjing, Nanjing 210009, People’s Republic of China

## Abstract

In the title compound, C_15_H_17_NO_8_, the nitro group is essentially coplanar with the aromatic ring [dihedral angle = 6.4 (3) Å]. The five-membered ring has a twist conformation. In the crystal, C—H⋯O inter­actions link the mol­ecules into a helical chain propagating along [010].

## Related literature

For the synthesis of the title compound, see: Kim *et al.* (1994 ▶). For the use of (2*S*,3S)-diethyl 2,3-*O*-alkyl­tartrate analogues as inter­mediates in organic synthesis, see: Pandey *et al.* (1997 ▶). For typical bond-length data, see: Allen *et al.* (1987 ▶).
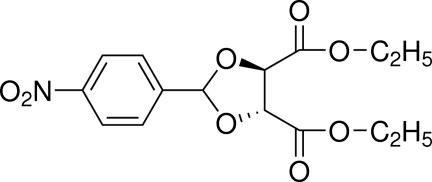


## Experimental

### 

#### Crystal data

C_15_H_17_NO_8_
*M* *_r_* = 339.30
Monoclinic, 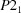

*a* = 12.261 (3) Å
*b* = 4.5200 (9) Å
*c* = 15.656 (3) Å
β = 112.27 (3)°
*V* = 802.9 (3) Å^3^
*Z* = 2
Mo *K*α radiation
μ = 0.12 mm^−1^
*T* = 293 K
0.30 × 0.20 × 0.10 mm

#### Data collection

Enraf-Nonius CAD-4 diffractometer
Absorption correction: ψ scan (North *et al.*, 1968 ▶) *T*_min_ = 0.966, *T*_max_ = 0.989
3062 measured reflections
1660 independent reflections
1364 reflections with *I* > 2σ(*I*)
*R*_int_ = 0.017
3 standard reflections every 200 reflections intensity decay: 1%

#### Refinement

*R*[*F* ^2^ > 2σ(*F* ^2^)] = 0.046
*wR*(*F* ^2^) = 0.142
*S* = 1.01
1660 reflections
218 parameters
1 restraint
H-atom parameters constrained
Δρ_max_ = 0.21 e Å^−3^
Δρ_min_ = −0.16 e Å^−3^

### 

Data collection: *CAD-4 Software* (Enraf–Nonius, 1989 ▶); cell refinement: *CAD-4 Software*; data reduction: *XCAD4* (Harms & Wocadlo, 1995 ▶); program(s) used to solve structure: *SHELXS97* (Sheldrick, 2008 ▶); program(s) used to refine structure: *SHELXL97* (Sheldrick, 2008 ▶); molecular graphics: *PLATON* (Spek, 2009 ▶); software used to prepare material for publication: *SHELXL97*.

## Supplementary Material

Crystal structure: contains datablock(s) global, I. DOI: 10.1107/S160053681201118X/su2384sup1.cif

Structure factors: contains datablock(s) I. DOI: 10.1107/S160053681201118X/su2384Isup2.hkl

Supplementary material file. DOI: 10.1107/S160053681201118X/su2384Isup3.cml

Additional supplementary materials:  crystallographic information; 3D view; checkCIF report

## Figures and Tables

**Table 1:** Hydrogen-bond geometry (Å, °)

*D*—H⋯*A*	*D*—H	H⋯*A*	*D*⋯*A*	*D*—H⋯*A*
C12—H12*A*⋯O8^i^	0.96	2.50	3.356 (7)	149

Symmetry code: (i) 
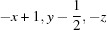
.

**Fig. 1.**
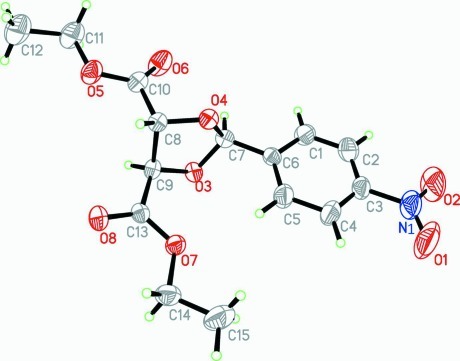
The molecular structure of the title compound showing the atom-numbering. Displacement ellipsoids are drawn at the 30% probability level.

**Fig. 2.**
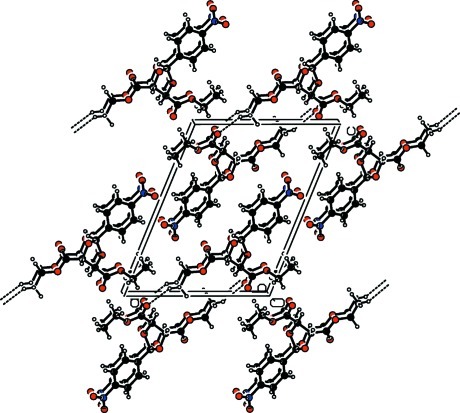
A view along the b axis of the crystal packing of the title compound, with the C-H···O interactions shown as dashed lines.

## supplementary crystallographic information

### Comment

Antitumor platinum drugs are one of the most effective anticancer agents currently available. (2*S*,3S)-Diethyl 2,3-*O*-alkyltartrate analogues are starting materials for the synthesis of platinum complexes with antitumor activity (Kim *et al.*, 1994), and they are also important intermediates in organic synthesis (Pandey *et al.*, 1997). As part of our studies of the synthesis and characterization of such compounds, we herein report on the crystal structure of the title compound.

The molecular structure of the title compound is shown in Fig. 1. The bond lengths are within normal ranges (Allen *et al.*, 1987). The nitro group (N1/O1/O2) is essentially coplanar with the aromatic ring (C1-C6) being inclined to it by 6.4 (3)°. The five-membered ring (O3/O4/C7-C9) has a twist conformation on bond O4-C8.

In the crystal, a C—H···O interaction (Table 1) links the molecules to form a a helical chain propagating along the b axis direction (Fig. 2).

### Experimental

4-Nitrobenzaldehyde (299 mg, 1.98 mmol), (2*S*,3S)-diethyltartrate (378 mg, 1.84 mmol) and cyclohexane (10 ml) were placed in a round-bottomed flask, and 30 mg of 4-methylbenzenesulfonic acid was added. The flask was fitted with a water distributor. The mixture was heated under reflux for 4 h. The reaction mixture was then added dropwise to water (600 ml) with vigorous stirring. A pale yellow precipitate was obtained, filtered off and dried in vacuo. Colourless block-like crystals, suitable for X-ray analysis, were obtained by slow evaporation of a methanol solution after 4 weeks.

### Refinement

The NH and C-bound H-atoms were included in calculated positions and treated as riding atoms: N-H = 0.86 Å, C-H = 0.93, 0.96, 0.97 and 0.98 Å for CH(aromatic), CH_3_, CH_2_ and CH(methine) H-atoms, respectively, with U_iso_(H) = k × U_eq_(N,C), where k = 1.5 for CH_3_ H-atoms and k = 1.2 for all other H-atoms. In the final cycles of refinement, in the absence of significant anomalous scattering effects, 1257 Friedel pairs were merged and Δf " set to zero.

### Crystal data

**Table:**

C_15_H_17_NO_8_	*F*(000) = 356
*M**_r_* = 339.30	*D*_x_ = 1.403 Mg m^−^^3^
Monoclinic, *P*2_1_	Mo *K*α radiation, λ = 0.71073 Å
Hall symbol: P 2yb	Cell parameters from 25 reflections
*a* = 12.261 (3) Å	θ = 9–13°
*b* = 4.5200 (9) Å	µ = 0.12 mm^−^^1^
*c* = 15.656 (3) Å	*T* = 293 K
β = 112.27 (3)°	Block, colourless
*V* = 802.9 (3) Å^3^	0.30 × 0.20 × 0.10 mm
*Z* = 2	

### Data collection

**Table:**

Enraf-Nonius CAD-4 diffractometer	1364 reflections with *I* > 2σ(*I*)
Radiation source: fine-focus sealed tube	*R*_int_ = 0.017
Graphite monochromator	θ_max_ = 25.3°, θ_min_ = 1.4°
ω/2θ scans	*h* = 0→14
Absorption correction: ψ scan (North *et al.*, 1968)	*k* = −5→5
*T*_min_ = 0.966, *T*_max_ = 0.989	*l* = −18→17
3062 measured reflections	3 standard reflections every 200 reflections
1660 independent reflections	intensity decay: 1%

### Refinement

**Table:**

Refinement on *F*^2^	Secondary atom site location: difference Fourier map
Least-squares matrix: full	Hydrogen site location: inferred from neighbouring sites
*R*[*F*^2^ > 2σ(*F*^2^)] = 0.046	H-atom parameters constrained
*wR*(*F*^2^) = 0.142	*w* = 1/[σ^2^(*F*_o_^2^) + (0.1*P*)^2^ + 0.070*P*] where *P* = (*F*_o_^2^ + 2*F*_c_^2^)/3
*S* = 1.01	(Δ/σ)_max_ < 0.001
1660 reflections	Δρ_max_ = 0.21 e Å^−^^3^
218 parameters	Δρ_min_ = −0.16 e Å^−^^3^
1 restraint	Extinction correction: *SHELXL97* (Sheldrick, 2008), Fc^*^=kFc[1+0.001xFc^2^λ^3^/sin(2θ)]^-1/4^
Primary atom site location: structure-invariant direct methods	Extinction coefficient: 0.036 (9)

### Special details

**Table:**

Geometry. All e.s.d.'s (except the e.s.d. in the dihedral angle between two l.s. planes) are estimated using the full covariance matrix. The cell e.s.d.'s are taken into account individually in the estimation of e.s.d.'s in distances, angles and torsion angles; correlations between e.s.d.'s in cell parameters are only used when they are defined by crystal symmetry. An approximate (isotropic) treatment of cell e.s.d.'s is used for estimating e.s.d.'s involving l.s. planes.
Refinement. Refinement of *F*^2^ against ALL reflections. The weighted *R*-factor *wR* and goodness of fit *S* are based on *F*^2^, conventional *R*-factors *R* are based on *F*, with *F* set to zero for negative *F*^2^. The threshold expression of *F*^2^ > σ(*F*^2^) is used only for calculating *R*-factors(gt) *etc*. and is not relevant to the choice of reflections for refinement. *R*-factors based on *F*^2^ are statistically about twice as large as those based on *F*, and *R*- factors based on ALL data will be even larger.

### Fractional atomic coordinates and isotropic or equivalent isotropic displacement parameters (Å^2^)

**Table:**

	*x*	*y*	*z*	*U*_iso_*/*U*_eq_	
N1	0.1597 (5)	0.1051 (17)	0.6035 (3)	0.1016 (18)	
C1	0.3496 (4)	−0.1471 (14)	0.4814 (3)	0.0770 (14)	
H1A	0.4154	−0.2670	0.4932	0.092*	
O1	0.0820 (6)	0.276 (2)	0.5904 (4)	0.163 (3)	
C2	0.3039 (4)	−0.1052 (17)	0.5489 (3)	0.0902 (18)	
H2A	0.3396	−0.1929	0.6066	0.108*	
O2	0.2028 (4)	−0.0395 (18)	0.6735 (3)	0.146 (3)	
O3	0.26487 (19)	−0.1203 (6)	0.23682 (14)	0.0483 (6)	
C3	0.2075 (4)	0.0631 (14)	0.5303 (3)	0.0703 (12)	
O4	0.40116 (19)	0.2240 (6)	0.31395 (14)	0.0465 (6)	
C4	0.1556 (4)	0.2006 (17)	0.4495 (3)	0.0924 (19)	
H4A	0.0895	0.3184	0.4387	0.111*	
O5	0.5315 (2)	0.1545 (8)	0.14859 (18)	0.0685 (8)	
C5	0.2020 (4)	0.1647 (15)	0.3817 (3)	0.0855 (17)	
H5A	0.1675	0.2616	0.3254	0.103*	
C6	0.2981 (3)	−0.0121 (9)	0.3972 (2)	0.0490 (9)	
O6	0.5589 (3)	−0.1270 (9)	0.2711 (2)	0.0884 (11)	
C7	0.3507 (3)	−0.0501 (8)	0.3259 (2)	0.0469 (8)	
H7A	0.4116	−0.2038	0.3455	0.056*	
C8	0.4003 (3)	0.2225 (8)	0.2239 (2)	0.0445 (8)	
H8A	0.3970	0.4254	0.2010	0.053*	
O7	0.1014 (2)	0.2755 (6)	0.15578 (16)	0.0555 (7)	
C9	0.2829 (3)	0.0569 (8)	0.1691 (2)	0.0452 (8)	
H9A	0.2942	−0.0709	0.1226	0.054*	
O8	0.1850 (2)	0.4257 (7)	0.05872 (16)	0.0650 (8)	
C10	0.5053 (3)	0.0613 (9)	0.2180 (3)	0.0525 (9)	
C11	0.6333 (4)	0.0176 (15)	0.1381 (3)	0.0895 (16)	
H11A	0.6229	−0.1952	0.1325	0.107*	
H11B	0.7040	0.0595	0.1917	0.107*	
C12	0.6445 (5)	0.1387 (18)	0.0547 (4)	0.107 (2)	
H12A	0.7100	0.0469	0.0457	0.161*	
H12B	0.6573	0.3484	0.0616	0.161*	
H12C	0.5735	0.1000	0.0022	0.161*	
C13	0.1838 (3)	0.2735 (8)	0.1218 (2)	0.0449 (8)	
C14	0.0027 (4)	0.4800 (12)	0.1144 (3)	0.0712 (12)	
H14A	0.0301	0.6610	0.0960	0.085*	
H14B	−0.0562	0.3910	0.0601	0.085*	
C15	−0.0484 (5)	0.5452 (19)	0.1824 (4)	0.117 (2)	
H15A	−0.1132	0.6799	0.1563	0.175*	
H15B	0.0103	0.6335	0.2359	0.175*	
H15C	−0.0761	0.3653	0.1998	0.175*	

### Atomic displacement parameters (Å^2^)

**Table:**

	*U*^11^	*U*^22^	*U*^33^	*U*^12^	*U*^13^	*U*^23^
N1	0.105 (3)	0.139 (5)	0.084 (3)	−0.026 (4)	0.062 (3)	−0.016 (3)
C1	0.071 (2)	0.095 (4)	0.068 (2)	0.019 (3)	0.030 (2)	0.030 (3)
O1	0.183 (5)	0.223 (8)	0.135 (4)	0.052 (7)	0.119 (4)	0.004 (5)
C2	0.093 (3)	0.125 (5)	0.056 (2)	0.000 (4)	0.032 (2)	0.028 (3)
O2	0.176 (4)	0.200 (7)	0.094 (2)	−0.031 (5)	0.089 (3)	0.015 (4)
O3	0.0611 (13)	0.0358 (12)	0.0482 (12)	−0.0079 (12)	0.0209 (10)	−0.0006 (10)
C3	0.073 (2)	0.091 (3)	0.056 (2)	−0.022 (3)	0.0355 (19)	−0.011 (2)
O4	0.0525 (12)	0.0379 (13)	0.0504 (12)	−0.0032 (11)	0.0208 (9)	−0.0071 (11)
C4	0.088 (3)	0.130 (5)	0.072 (3)	0.036 (4)	0.045 (2)	0.007 (4)
O5	0.0702 (16)	0.073 (2)	0.0773 (16)	0.0163 (16)	0.0448 (13)	0.0028 (16)
C5	0.090 (3)	0.112 (5)	0.060 (2)	0.045 (3)	0.036 (2)	0.021 (3)
C6	0.0488 (17)	0.048 (2)	0.0468 (17)	−0.0029 (17)	0.0141 (14)	0.0011 (16)
O6	0.090 (2)	0.078 (2)	0.117 (2)	0.034 (2)	0.0619 (19)	0.033 (2)
C7	0.0506 (17)	0.0374 (18)	0.0508 (17)	0.0003 (16)	0.0169 (14)	0.0011 (15)
C8	0.0494 (17)	0.0367 (17)	0.0495 (16)	−0.0036 (16)	0.0212 (14)	−0.0032 (16)
O7	0.0547 (13)	0.0584 (17)	0.0582 (13)	0.0070 (13)	0.0267 (11)	0.0119 (13)
C9	0.0597 (19)	0.0338 (17)	0.0486 (16)	−0.0033 (16)	0.0279 (15)	−0.0046 (15)
O8	0.0735 (16)	0.071 (2)	0.0550 (13)	0.0023 (16)	0.0290 (12)	0.0172 (15)
C10	0.058 (2)	0.043 (2)	0.062 (2)	−0.0007 (19)	0.0291 (17)	−0.0052 (19)
C11	0.090 (3)	0.095 (4)	0.109 (3)	0.022 (3)	0.066 (3)	−0.007 (3)
C12	0.112 (4)	0.124 (6)	0.118 (4)	0.014 (4)	0.081 (3)	−0.009 (4)
C13	0.0490 (17)	0.0425 (19)	0.0401 (15)	−0.0088 (15)	0.0135 (14)	−0.0045 (15)
C14	0.061 (2)	0.073 (3)	0.078 (3)	0.017 (2)	0.0242 (19)	0.014 (2)
C15	0.100 (4)	0.136 (7)	0.133 (4)	0.057 (5)	0.065 (3)	0.027 (5)

### Geometric parameters (Å, º)

**Table:**

N1—O1	1.183 (9)	C7—H7A	0.9800
N1—O2	1.212 (8)	C8—C10	1.512 (5)
N1—C3	1.483 (6)	C8—C9	1.560 (4)
C1—C6	1.370 (5)	C8—H8A	0.9800
C1—C2	1.384 (6)	O7—C13	1.309 (4)
C1—H1A	0.9300	O7—C14	1.465 (5)
C2—C3	1.341 (8)	C9—C13	1.517 (5)
C2—H2A	0.9300	C9—H9A	0.9800
O3—C9	1.411 (4)	O8—C13	1.208 (4)
O3—C7	1.428 (4)	C11—C12	1.470 (7)
C3—C4	1.335 (7)	C11—H11A	0.9700
O4—C8	1.406 (4)	C11—H11B	0.9700
O4—C7	1.428 (4)	C12—H12A	0.9600
C4—C5	1.391 (6)	C12—H12B	0.9600
C4—H4A	0.9300	C12—H12C	0.9600
O5—C10	1.314 (5)	C14—C15	1.455 (7)
O5—C11	1.458 (5)	C14—H14A	0.9700
C5—C6	1.367 (6)	C14—H14B	0.9700
C5—H5A	0.9300	C15—H15A	0.9600
C6—C7	1.497 (5)	C15—H15B	0.9600
O6—C10	1.197 (5)	C15—H15C	0.9600
			
O1—N1—O2	123.7 (5)	O3—C9—C13	114.1 (3)
O1—N1—C3	118.6 (6)	O3—C9—C8	103.5 (2)
O2—N1—C3	117.7 (6)	C13—C9—C8	111.1 (3)
C6—C1—C2	120.0 (5)	O3—C9—H9A	109.3
C6—C1—H1A	120.0	C13—C9—H9A	109.3
C2—C1—H1A	120.0	C8—C9—H9A	109.3
C3—C2—C1	119.4 (4)	O6—C10—O5	124.1 (4)
C3—C2—H2A	120.3	O6—C10—C8	123.8 (3)
C1—C2—H2A	120.3	O5—C10—C8	112.1 (3)
C9—O3—C7	109.9 (3)	O5—C11—C12	108.3 (4)
C4—C3—C2	122.3 (4)	O5—C11—H11A	110.0
C4—C3—N1	119.1 (5)	C12—C11—H11A	110.0
C2—C3—N1	118.6 (5)	O5—C11—H11B	110.0
C8—O4—C7	106.7 (3)	C12—C11—H11B	110.0
C3—C4—C5	118.9 (5)	H11A—C11—H11B	108.4
C3—C4—H4A	120.6	C11—C12—H12A	109.5
C5—C4—H4A	120.6	C11—C12—H12B	109.5
C10—O5—C11	116.5 (4)	H12A—C12—H12B	109.5
C6—C5—C4	120.4 (4)	C11—C12—H12C	109.5
C6—C5—H5A	119.8	H12A—C12—H12C	109.5
C4—C5—H5A	119.8	H12B—C12—H12C	109.5
C5—C6—C1	118.9 (4)	O8—C13—O7	125.3 (3)
C5—C6—C7	121.4 (3)	O8—C13—C9	120.8 (3)
C1—C6—C7	119.7 (3)	O7—C13—C9	113.9 (3)
O4—C7—O3	105.1 (3)	C15—C14—O7	108.5 (4)
O4—C7—C6	109.2 (3)	C15—C14—H14A	110.0
O3—C7—C6	112.8 (3)	O7—C14—H14A	110.0
O4—C7—H7A	109.9	C15—C14—H14B	110.0
O3—C7—H7A	109.9	O7—C14—H14B	110.0
C6—C7—H7A	109.9	H14A—C14—H14B	108.4
O4—C8—C10	112.4 (3)	C14—C15—H15A	109.5
O4—C8—C9	102.0 (2)	C14—C15—H15B	109.5
C10—C8—C9	111.4 (3)	H15A—C15—H15B	109.5
O4—C8—H8A	110.3	C14—C15—H15C	109.5
C10—C8—H8A	110.3	H15A—C15—H15C	109.5
C9—C8—H8A	110.3	H15B—C15—H15C	109.5
C13—O7—C14	117.5 (3)		
			
C6—C1—C2—C3	1.4 (9)	C7—O4—C8—C10	−83.6 (3)
C1—C2—C3—C4	−2.1 (10)	C7—O4—C8—C9	35.8 (3)
C1—C2—C3—N1	179.9 (6)	C7—O3—C9—C13	−114.2 (3)
O1—N1—C3—C4	−5.3 (10)	C7—O3—C9—C8	6.7 (3)
O2—N1—C3—C4	174.5 (6)	O4—C8—C9—O3	−25.9 (3)
O1—N1—C3—C2	172.7 (7)	C10—C8—C9—O3	94.2 (3)
O2—N1—C3—C2	−7.5 (9)	O4—C8—C9—C13	97.1 (3)
C2—C3—C4—C5	0.9 (10)	C10—C8—C9—C13	−142.9 (3)
N1—C3—C4—C5	178.9 (6)	C11—O5—C10—O6	−0.6 (6)
C3—C4—C5—C6	1.0 (10)	C11—O5—C10—C8	178.6 (4)
C4—C5—C6—C1	−1.6 (9)	O4—C8—C10—O6	24.7 (5)
C4—C5—C6—C7	−179.0 (5)	C9—C8—C10—O6	−89.0 (4)
C2—C1—C6—C5	0.5 (8)	O4—C8—C10—O5	−154.5 (3)
C2—C1—C6—C7	177.8 (5)	C9—C8—C10—O5	91.7 (4)
C8—O4—C7—O3	−32.8 (3)	C10—O5—C11—C12	177.1 (5)
C8—O4—C7—C6	−154.0 (2)	C14—O7—C13—O8	−0.4 (5)
C9—O3—C7—O4	14.8 (3)	C14—O7—C13—C9	179.6 (3)
C9—O3—C7—C6	133.7 (3)	O3—C9—C13—O8	−175.6 (3)
C5—C6—C7—O4	67.0 (5)	C8—C9—C13—O8	67.8 (4)
C1—C6—C7—O4	−110.3 (4)	O3—C9—C13—O7	4.4 (4)
C5—C6—C7—O3	−49.4 (5)	C8—C9—C13—O7	−112.2 (3)
C1—C6—C7—O3	133.3 (4)	C13—O7—C14—C15	−155.4 (5)

### Hydrogen-bond geometry (Å, º)

**Table:**

*D*—H···*A*	*D*—H	H···*A*	*D*···*A*	*D*—H···*A*
C12—H12*A*···O8^i^	0.96	2.50	3.356 (7)	149

Symmetry code: (i) −*x*+1, *y*−1/2, −*z*.

## References

[bb1] Allen, F. H., Kennard, O., Watson, D. G., Brammer, L., Orpen, A. G. & Taylor, R. (1987). *J. Chem. Soc. Perkin Trans. 2*, pp. S1–19.

[bb2] Enraf–Nonius (1989). *CAD-4 Software* Enraf–Nonius, Delft, The Netherlands.

[bb3] Harms, K. & Wocadlo, S. (1995). *XCAD4* University of Marburg, Germany.

[bb4] Kim, D. K., Kim, G., Gam, J. S., Cho, Y. B. & Park, J. G. (1994). *J. Med. Chem.* **37**, 147–1485. 10.1021/jm00036a013 8182706

[bb5] North, A. C. T., Phillips, D. C. & Mathews, F. S. (1968). *Acta Cryst.* A**24**, 351–359.

[bb6] Pandey, G., Hajra, S., Ghorai, M. K. & Kumar, K. R. (1997). *J. Org. Chem.* **62**, 5966–5973.

[bb7] Sheldrick, G. M. (2008). *Acta Cryst.* A**64**, 112–122. 10.1107/S0108767307043930 18156677

[bb8] Spek, A. L. (2009). *Acta Cryst.* D**65**, 148–155. 10.1107/S090744490804362X PMC2631630 19171970

